# Prediction of models for ordered solvent in macromolecular structures by a classifier based upon resolution-independent projections of local feature data

**DOI:** 10.1107/S2059798319008933

**Published:** 2019-07-30

**Authors:** Laurel Jones, Michael Tynes, Paul Smith

**Affiliations:** a Department of Chemistry, Fordham University, Bronx, NY 10458, USA; b Department of Computer and Information Science, Fordham University, Bronx, NY 10458, USA

**Keywords:** *PeakProbe*, solvent modelling, electron-density analysis, supervised learning, decorrelation, resolution, data mining

## Abstract

Current software tools for the automated building of models for macro­molecular X-ray crystal structures are capable of assembling high-quality models for ordered macromolecule and small-molecule scattering components with minimal or no user supervision. Many of these tools also incorporate robust functionality for modelling the ordered water molecules that are found in nearly all macromolecular crystal structures. However, no current tools focus on differentiating these ubiquitous water molecules from other frequently occurring multi-atom solvent species, such as sulfate, or the automated building of models for such species. *PeakProbe* has been developed specifically to address the need for such a tool. *PeakProbe* predicts likely solvent models for a given point (termed a ‘peak’) in a structure based on analysis (‘probing’) of its local electron density and chemical environment. *PeakProbe* maps a total of 19 resolution-dependent features associated with electron density and two associated with the local chemical environment to a two-dimensional score space that is independent of resolution. Peaks are classified based on the relative frequencies with which four different classes of solvent (including water) are observed within a given region of this score space as determined by large-scale sampling of solvent models in the Protein Data Bank. Designed to classify peaks generated from difference density maxima, *PeakProbe* also incorporates functionality for identifying peaks associated with model errors or clusters of peaks likely to correspond to multi-atom solvent, and for the validation of existing solvent models using solvent-omit electron-density maps. When tasked with classifying peaks into one of four distinct solvent classes, *PeakProbe* achieves greater than 99% accuracy for both peaks derived directly from the atomic coordinates of existing solvent models and those based on difference density maxima. While the program is still under development, a fully functional version is publicly available. *PeakProbe* makes extensive use of *cctbx* libraries, and requires a *PHENIX* licence and an up-to-date *phenix.python* environment for execution.

## Abstract 2

*PeakProbe* facilitates the automated modelling of ordered solvent in macromolecular crystal structures by analysing features of the electron density and chemical environment surrounding a given coordinate. The extracted data are transformed to a resolution-independent score space and likely solvent models are predicted based on the frequency distributions observed in a large-scale sample of the PDB.

## 1. Introduction   

Current techniques in macromolecular X-ray crystallography derive structural information by the construction of a comprehensive model of X-ray scattering components within a crystal system. Besides the integral nucleic and amino-acid polymers found in macromolecular structures, other scattering components include ligands and cofactors associated with these polymers, and both ordered/explicit and bulk solvent. Crystallographic model building relies on reconstructing electron density from Fourier coefficients whose complex components (phases) are ultimately derived in whole or in large part by Fourier transformation of a model of all scattering components. As a result, crystallographic models are best built through an iterative process in which more complete and accurate models result in more accurate phases which provide sharper and more interpretable electron density, which in turn allows the assembly of a further improved model, further improved phases and so on. Thus, arriving at an optimal model for a crystal structure requires the inclusion of all scattering components, including the often numerous ordered small-molecule and solvent species within the crystal lattice (Drenth & Mesters, 2007 ▸).

Water molecules are by far the most frequently modelled non-macromolecular species in macromolecular structures, followed by oxyanions, organic polymers and atomic ions. Specifically, a survey of 111 976 X-ray structures from the Protein Data Bank (PDB; as of 29 June 2017) revealed that 93% of deposited structures include explicit water molecules, with an average of 320 water molecules per structure (over 32 million in total), with one water modelled for every 13 non-H macromolecular atoms (Berman *et al.*, 2000 ▸; Gnesi & Carugo, 2017 ▸). After water, the tetrahedral oxyanions SO_4_^2−^ and PO_4_^3−^ are the next most frequent small-molecule species modelled, with 76 500 such molecules distributed among 18% of all structures. Individually less frequent but collectively relatively common are polyatomic species such as acetate ions, glycerol and various lengths of polyethylene glycol (PEG) polymer and atomic ions such as Mg^2+^ and Zn^2+^, which occur in 22% and 25% of structures, respectively. For convenience, we refer to these species collectively as ‘solvent’ species, along with any similar molecules that are not either covalently associated with a macromolecule or an integral macromolecular cofactor (*e.g.* heme).

Solvent species arise in macromolecular structures from various sources. Common sources include the chemical environment used to grow the macromolecular crystals and the solution components in which the macromolecules themselves are isolated. However, solvent species can appear in structures as a result of carry-over from upstream purification or contamination from common laboratory reagents (Niedzialkowska *et al.*, 2016 ▸; Das *et al.*, 2012 ▸). Thus, a list of chemical species that are explicitly present during crystal growth does not provide an exhaustive list of chemical species that should be considered when model building. Ultimately, ideal atomic models for any crystal structure should incorporate appropriate coordinates for all ordered species, regardless of their origin.

Many software tools exist for automated model building of both the macromolecular and solvent components of macromolecular structures. To one degree or another, all approaches to automated modelling employ fundamental aspects of artificial intelligence (AI), a topic that has been associated with crystallography for over 40 years (Feigenbaum *et al.*, 1977 ▸). In addition to the mainstay AI techniques of knowledge representation and decision making, many programs for automated model building implement supervised machine learning in which knowledge about the features associated with a set of characterized instances is leveraged to make predictions about uncharacterized instances (Morshed *et al.*, 2015 ▸). Successful applications of this approach to the problem of modelling peptide/protein structures include pattern matching of templates of known protein structural motifs to electron density [*RESOLVE*/*PHENIX* (Terwilliger, 2001 ▸; Adams *et al.*, 2010 ▸), *Buccaneer* (Cowtan, 2006 ▸)], extraction of local electron-density features followed by analysis by a trained classifier (*TEXTAL*; Holton *et al.*, 2000 ▸) and a mixture of these approaches that entails matching the patterns of likely atomic positions to probability distributions of atomic arrangements found in the PDB (*ARP*/*wARP*; Morris *et al.*, 2003 ▸). Additional routines from these and other software packages provide functionality for building nucleic acid structures, peptide loop fitting/building and rotamer identification, and rebuilding incorrect, incomplete or divergent models such as those derived from molecular-replacement solutions. While not yet ‘self-driving’, automated macromolecular modelling can produce structures that often match and sometimes surpass in quality those built ‘by hand’ in terms of stereochemical outliers and agreement with electron density (Joosten *et al.*, 2009 ▸).

Similarly, numerous computational approaches have been implemented to automate the building of solvent species. In broad terms, current tools for modelling solvent species focus on either building water models globally for an entire structure or identifying and modelling specific small-molecule species within specific areas of electron density. In contrast, the *PeakProbe* software that we describe has been designed with the express purpose of identifying what solvent model, if any, is likely to exist at a given coordinate within a macromolecular structure. Below, we detail common approaches to automated solvent model building and the limitations of current tools that motivated the development of *PeakProbe*.

### 1.1. Solvent-modelling capabilities of current automated structure-building tools   

The problem of modelling solvent species naturally bifurcates into modelling single-atom versus multi-atom solvent species. Single-atom species include the ubiquitous water (H atoms are ignored), common elemental anions such as chloride, and both monovalent and divalent metal ions. Because all of these species can be modelled as a single point, building a correct model for a single atom only requires determining which, if any, species should be placed at a given point of density, followed by crystallographic refinement of coordinates and *B* factors. In contrast, automated modelling of multi-atom species requires significantly more effort owing to both the diversity and the complexity of such species. Among the 100 most encountered solvent species in the PDB, only 17 (including water) are single-atom species and roughly 1000 unique multi-atom species have ten or more instances in the PDB. Furthermore, the majority of multi-atom species exhibit multiple distinct conformers. Thus, the sheer spectrum of possible multi-atom solvent species, compounded by the need to explore multiple conformations when evaluating possible solvent candidates, exponentiates the complexity of automating the model-building process. Many highly capable tools exist for single-atom modelling and, despite the difficulty of the task, much progress has been made towards automating the modelling of multi-atom species.

The current automated approaches to modelling single-atom species all employ some variant of a two-step procedure involving first identifying positions for potential solvent molecules followed by the evaluation of each position with respect to target parameters. For example, one of the earliest and still most common approaches for water modelling entails identifying potential water positions from peaks in a difference density (*F*_o_ − *F*_c_) map followed by the evaluation of each position with respect to hydrogen-bond donors/acceptors and crystallographic *B* factors following model refinement [*ASIR* (Tong *et al.*, 1994 ▸), *DDQ* (van den Akker & Hol, 1999 ▸)]. Automated modelling of nonwater single-atom species, such as elemental ions, extends this procedure by incorporating the evaluation of properties such as coordination geometry, anomalous scattering and valence-bond character that differ between ions and water and between different ions [*SHELXL* (Müller *et al.*, 2003 ▸), *PHENIX* (Echols *et al.*, 2014 ▸)]. Importantly, such tools only provide models for single-atom species and do not provide for the modelling of common multi-atom solvent species such as sulfate.

*PeakProbe* was designed to avoid model errors caused by incorrectly modelling multi-atom solvent as a cluster of water molecules. Indeed, a random assortment of ‘free atoms’ can be arranged and refined to faithfully recapitulate all density within a given unit cell, a procedure that is at the core of the powerful phase-improvement and model-building algorithms in *ARP*/*wARP* (Perrakis *et al.*, 1999 ▸). Consequently, given permissive and persistent refinement of positions and *B* factors, a collection of waters can convincingly serve as a model for a multi-atom solvent molecule. Ideally, tools for the automated modelling of explicit solvent would avoid such errors by discriminating between points where single-atom models are appropriate and those where multi-atom solvent should be considered. With this goal in mind, we have developed *PeakProbe* with a particular focus on leveraging features that distinguish water from nonwater solvent species.

The current tools for modelling multi-atom species are all well equipped to model simple rigid (*i.e.* without rotatable bonds) molecules such as sulfate as well as conformationally complex ligands (*e.g.* adenosine triphosphate). One approach for multi-atom solvent modelling includes the placement of fragment models by a brute-force search, followed by the conformational sampling of molecules homologous to this fragment to maximize the fit to electron density (*PHENIX*; Terwilliger *et al.*, 2006 ▸). Another approach involves orienting candidate molecules within a region of electron density by the alignment of model and electron-density inertia tensors (*X-LIGAND*; Oldfield, 2001 ▸) or the eigenvectors of centred distance matrices (*Coot*; Emsley & Cowtan, 2004 ▸) followed by sampling of conformational space by either statistical sampling techniques (for example Monte Carlo) or brute force. Yet another approach uses distance-based graph/subgraph isomorphism to successively place atoms from a candidate model at points within the electron density identified as likely atoms, accounting for torsional flexibility as successive atoms are placed (*X-LIGAND*, *ARP*/*wARP*). However, the multi-atom modelling capabilities of each of these programs are geared towards evaluating one or more candidate molecules at either user-defined locations or within large volumes of unmodelled electron density. This approach is not easily adapted to determining what single- or multi-atom species, if any, best occupies a given point or to quickly evaluate all likely solvent positions within a structure. Thus, we have designed *Peak­Probe* to operate with no prior knowledge or expectations about a given structure and to work sufficiently quickly for practical evaluation of all positions within a structure that are likely to be associated with a solvent model.

Because the crystallographic resolution inherently reflects the amount of information contained within any region of electron density available for predicting a correct model, the performance of all automated modelling approaches suffers as resolution worsens (Luzzati, 1952 ▸). Approaches that rely upon the accurate placement of candidate atoms as starting points for model building, such as *ARP*/*wARP*, become less accurate at resolutions where errors in the placement of such candidates obfuscate subsequent pattern-matching routines (typically >2.7 Å). Pattern-matching approaches such as those used in *RESOLVE* and *Buccaneer* can, but do not always, fare better at these resolutions. Likewise, solvent modelling becomes more challenging as resolution worsens and electron density becomes more amorphous. Analysis of PDB data suggests that the number of water molecules observed per amino acid drops by a factor of about five when comparing structures refined at 2.0 Å resolution with those at 3.0 Å resolution (Weichenberger *et al.*, 2015 ▸). The marked but unsurprising scarcity of structures at >3.0 Å resolution obfuscates detailed analysis of other single- or multi-atom species at these resolutions. Nonetheless, a comprehensive solvent-modelling program should attempt to correctly build solvent species at these resolutions where fully justified by observed data, even if such species are rare. Accordingly, we have employed various resolution-dependent data-processing schemes in *PeakProbe* to provide robust means of predicting correct solvent models over a wide range of crystallographic resolutions.

Modelling of solvent species involves the analysis of specific features of the electron density and atomic environment associated with a given point within a structure and deciding what model, if any, is most consistent with these features. Whether performed manually or via automation, choosing an appropriate model for a given location can make use of both empirical and theoretical considerations. For example, a putative water model would be expected to be associated with electron density similar to empirical density for known water molecules in structures obtained at a similar crystallographic resolution. Likewise, a candidate water model might be discarded if found to overlap with another atom to an extent deemed impossible by theoretical constraints. For modelling macromolecular components, *ARP*/*wARP* uses distributions of geometry observed in the PDB for scoring putative main-chain fragments, while many protein-validation programs compare observed backbone dihedral angles with theoretical values derived from the physical and chemical properties of l-peptides, such as those developed by Ramachandran *et al.* (1963 ▸).

In machine learning, classifiers are models that assign a class to a given input based on information about the input in the form of numerical features. A successful classifier requires features whose values take on distinct or distinguishable distributions for each possible output class. Training a classifier refers to developing and optimizing a mathematical model that relates a subset or range of feature values to specific classes. Such models can be generated from theoretical considerations or developed from empirical studies of examples where both feature values and class membership are known. Several software tools employ such trained classifiers for evaluating and predicting water models. Examples include the *EDIA*/*ProteinPlus* package, *WaterScore*, *WaterRank* and *WaterDock*. Using varied approaches, these programs employ classifiers trained using features extracted from a thoroughly curated subset of water models considered to be of high quality in the PDB (Nittinger *et al.*, 2015 ▸; Ross *et al.*, 2012 ▸; Lippert & Rarey, 2009 ▸; Amadasi *et al.*, 2008 ▸; García-Sosa *et al.*, 2003 ▸; reviewed in Nittinger *et al.*, 2018 ▸). The features used for classification in these programs have found use in characterizing solvent models other than water (Meyder *et al.*, 2017 ▸). For example, the models of metal-ion bond valency established by *SHELXL* provide a theoretical basis for features that *CheckMyMetal* and *phenix.refine* use to distinguish water from metal ions or to distinguish between different metal ions (Zheng *et al.*, 2014 ▸). Inspired by the demonstrated utility of the supervised learning approaches used by these programs, we have based the scoring metrics of *PeakProbe* on probabilistic models obtained from large-scale data mining of common solvent species found in the PDB.

## 2. *PeakProbe* program description   

The central design of *PeakProbe* focuses on the prediction of a likely solvent model at a given coordinate in a crystal system. Predictions are made by evaluating features extracted from the electron density and local atomic environment of a given point (termed a ‘peak’) and comparing the extracted values with observed distributions. As we conceived *PeakProbe* to be used during the building as well as the validation stages of modelling, we designed the program specifically with the evaluation (‘probing’) of difference map peaks in mind, thus the name ‘*PeakProbe*’. Our initial goal was to develop a classifier to distinguish between water and sulfate/phosphate, the next most common solvent species in the PDB after water. Crystallographic methods are not well suited to differentiating between sulfate and phosphate, so we consider the two to be indistinguishable and refer to them collectively as ‘sulfate’ hereafter. The core of the *PeakProbe* classifier uses two scores that encapsulate the overall sulfate-like nature of the local electron density and the chemical environment of a peak. Taken together, these two scores are able to discriminate between other types of solvent apart from water and sulfate. Specifically, the *PeakProbe* classifier has been trained to distinguish four classes of solvent: water, sulfate, heterogen and metal. The heterogen class includes other common solvent species with polar or anionic character such as PEG, glycerol, and acetate and chloride ions. The metal class refers specifically to divalent metals such as Mg^2+^, Ca^2+^, Zn^2+^ and Mn^2+^.

Our approach to development focused on three specific goals: (i) the program should predict solvent models that are highly consistent with existing models in the PDB with respect to both local electron density and chemical environments, (ii) the program should make such consistent predictions regardless of crystallographic resolution and (iii) the program should be capable of making such predictions at peaks that are not associated with any existing solvent model, such as those situated at difference density maxima.

To address these goals, we developed *PeakProbe* by assembling solvent models for four distinct classes of solvent, extracting various candidate classifier features from these models, evaluating these features based on their ability to discriminate between solvent species, constructing a data-processing pipeline to allow resolution-independent evaluation of features and implementing a classifier based on composite scores derived from feature data. We designed the feature extraction in *PeakProbe* to maximize the equivalence between features extracted from peaks whose coordinates were taken from the coordinates of existing solvent model atoms (solvent model peaks) and those extracted from peaks located at difference density maxima (difference density/map peaks). In addition, *PeakProbe* also makes use of extracted features to identify common model errors such as missing alternate conformations or incorrect rotamers (Lunin *et al.*, 2002 ▸). The cluster-analysis algorithms within *PeakProbe* differentiate between tightly associated groups of water molecules and peak clusters that are possibly associated with multi-atom solvent species.

**Figure 1:**
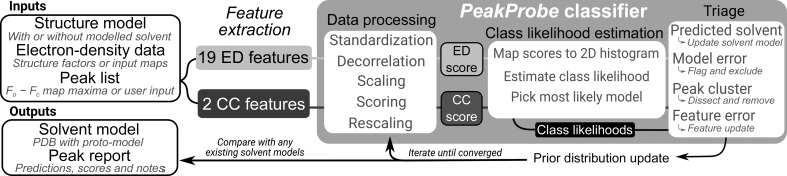
An overview of the *PeakProbe* structure and workflow.

As shown in Fig. 1 ▸, *PeakProbe* input consists of a macromolecular structure model, the corresponding structure-factor or map data and a peak list that specifies the coordinates to be evaluated. For each peak, a total of 21 features are extracted, 19 of which are associated with the local electron density of the peak (ED features). The two other features are derived from the local chemical environment of the peak (CC features). After extraction, these feature data are fed to the *PeakProbe* classifier, which implements three stages of analysis. In the first stage, ED and CC feature values are mapped to an ED and a CC score, respectively. In the second stage, the ED and CC scores are used to estimate the likelihood that the peak is a member of each class of solvent model. In the third stage, class likelihoods, ED and CC scores, and data on adjacent peaks and other models are used to categorize and triage each peak. During triage, a peak may be identified as (i) a possible solvent-model location, (ii) a result of model error, (iii) a member of a cluster of peaks or (iv) misclassified based on inconsistent feature values. Following triage, a list of peaks that are likely to correspond to solvent models is output or updated, peaks that are likely to be model errors are flagged, peak clusters are analysed and features are updated where appropriate. After these three steps, the frequency with which each class of solvent is observed in the output solvent model is used to update the prior distributions used by the classifier. The peaks are then reclassified using this updated prior and the entire classification process is repeated until no further changes are made to the accumulated list of predicted solvent models. Lastly, *PeakProbe* outputs a report on each peak that contains the results of the triage process, all score values, class likelihoods and a solvent-model prediction. If the initial structure model input contains existing solvent models, *PeakProbe* can evaluate coordinates from these models as peaks and can compare scores and predictions from existing models associated with input peaks when determining a final solvent model for each peak.

In its current configuration, *PeakProbe* will classify any type of peak input regardless of whether the peaks are derived from coordinates of existing solvent models, from difference map maxima or from user input. In addition, *PeakProbe* can perform pairwise comparison of difference map and solvent model peaks to validate/update existing models, while simultaneously identifying and classifying difference map peaks that are not associated with any existing model. By default, when given a structure containing a solvent model, *PeakProbe* removes all water, sulfate and other common solvent species and then calculates density maps and places peaks for analysis at local maxima of the resulting difference density map. Concurrently, coordinates for all solvent model atoms in the input structure are included as peaks provided that they were omitted during map calculation and occur less than 2.0 Å from a difference map peak. When determining likely solvent models for a given peak, *PeakProbe* also evaluates the properties of all adjacent peaks, including those input as solvent models, in order to determine the most likely model or models for all associated peaks. For example, an input structure may include two water models for a single difference map peak, or a given difference map peak may not be associated with any solvent model. Furthermore, multiple peaks may correspond to a single multi-atom solvent species. Regardless of the situation, *PeakProbe* evaluates the associations between all available peaks when determining the most likely model or models for a given peak.

In practice, *PeakProbe* selects the solvent class (water, sulfate, heterogen or metal) with the highest estimated likelihood as the predicted solvent model for each input peak. *PeakProbe* also outputs a PDB file containing the solvent models that the program considers to be both plausible and most likely for all input peaks. To generate coordinates for these models, *PeakProbe* either fits a candidate member of the predicted solvent class or carries over the solvent model from the input structure, provided that such a model was input and agrees with the predictions of the program. The *PeakProbe* classifier cannot yet distinguish between members of the heterogen or metal classes. Similarly, sulfate-like solvent species of compounds that contain a sulfate-like atomic centre such as cacodylate, citrate or 2-(*N*-morpholino)ethanesulfonic acid (MES) are likely to be classified as members of the sulfate class. Thus, the model-building capabilities of the program are limited and any multi-atom solvent model suggested by *PeakProbe* should be validated externally before inclusion in the complete structure model.

*PeakProbe* consists of ∼8000 lines of Python code and relies on numerous modules of the *cctbx* package for managing structure-data input/output, electron-density map generation, peak detection and coordinate refinement. All code is publicly available via GitHub along with documentation on software usage, software prerequisites and licence requirements (https://github.com/paulsmith638/PeakProbe.git).

### 2.1. Feature engineering   

To construct, train and evaluate our desired classifier, we assembled three data sets: (i) training data consisting of features extracted from peaks whose coordinates are taken exactly from the atomic positions of existing models in the PDB, (ii) validation data consisting of extracted features for peaks identical to those in the training data but including peaks derived from a broader range of existing solvent models and (iii) a testing data set of difference map peaks with which an existing solvent model could be clearly associated. We intended to base our classifier on the frequency with which a given set of feature values is observed for a given solvent class within a large subset of the entire PDB rather than for a particular subset of cherry-picked structures. To ensure adequate sampling of solvent models while keeping the computational overhead within reason, we elected to estimate global feature-value distributions based on distributions of values observed from a large set of structures varying widely in resolution, composition and quality. Comprehensive details for the curation and composition of each data set are given in Appendix *A* [App appa].

Our search for features that are likely to take on distinct distributions for water and sulfate focused both on the electron-density morphology and the disposition of atoms adjacent to models of each of these solvent species. To assemble candidate features related to electron density, we built upon several intuitive notions: (i) no matter how well refined, a sulfate model should yield a poor fit to density at a true water position and *vice versa*, (ii) the volume of electron density associated with a sulfate should be larger than that for water, (iii) for a peak centred at a given local density maximum corresponding to either water or the central sulfur of sulfate, density values for sulfate should fall off with radial distance less rapidly than for water and (iv) given sufficient resolution, the electron density of sulfate should appear tetrahedral. To quantify these notions for use as features, reference coordinate models for both sulfate and water were fitted to local electron density using real-space refinement. Following the refinement of each model at a given peak, a total of 19 numerical electron-density features were extracted (ED features; Table 1 ▸).

Of these features, 14 are real-space correlation coefficients (RSCC) for a given model and a given density map (Diamond, 1971 ▸). Using various combinations of input models and target electron densities yielded the 14 RSCC values described in Table 1 ▸ (CC1–CC14). In Table 1 ▸, the ‘Model’ and ‘Map’ columns refer to the coordinate model and density map used for RSCC calculation. The ‘Ref’ column indicates whether or not the coordinate model underwent real-space refinement prior to RSCC calculation. For example, CC4 corresponds to the RSCC for a sulfate model refined against the 2*F*_o_ − *F*_c_ density map. Maps marked with a ‘+’ refer to a pseudoinverse density as described in Appendix *A* [App appa]. Two additional features were statistically derived from these 14 RSCC values (ED1 and ED2). Features ED3 and ED4 correspond to peak size quantified by the volume of electron density surrounding a peak in both 2*F*_o_ − *F*_c_ and *F*_o_ − *F*_c_ density maps above a fixed contour level. Finally, feature ED5 is the 2*F*_o_ − *F*_c_ density value at the peak location itself.

To identify features of the local atomic environment that are distinct for water and sulfate, we investigated interatomic relationships between each peak within the training data and adjacent macromolecular and solvent atoms. When comparing relationships between sulfate and water, the two most systematically distinct features were the observed distance between a given peak and its closest neighbour atom and the preference of sulfate for electropositive over electronegative neighbour atoms. Both of these trends were expected because of the chemical properties of sulfate. Specifically, the central atom of sulfate (used as the peak for training) is buried within the molecule and does not form direct interactions with its neighbours. Thus, the distribution of distances between peaks at such positions and neighbouring model atoms should be systematically higher than that for water, which interacts directly with atomic neighbours. Accordingly, we employ the distance from a given peak to its nearest neighbour as a close contact feature (CF1). Similarly, as an anion, sulfate forms favourable electrostatic interactions with positively charged moieties such as arginine and lysine side chains and so would be expected to be observed more frequently in proximity to these functional groups and less frequently next to negatively charged groups such as aspartate and glutamate side chains. We quantify this propensity with a likelihood metric based on the relative observed frequencies of occurrence for certain macromolecular atoms near sulfate versus water (CF2; detailed in Appendix *A* [App appa]). In conjunction with the aforementioned 19 electron-density features, these two close-contact features (CC features) constitute the 21 total features used for classification. The observed distributions for each of the 21 feature extracted from training peaks were unimodal and resembled normal distributions with perceptible but not excessive skew or kurtosis.

### 2.2. Classifier design and construction   

**Figure 2:**
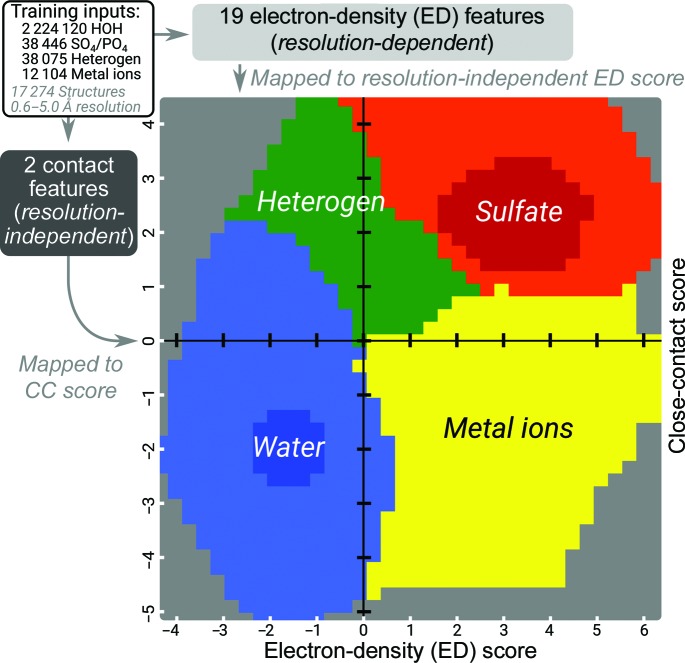
Solvent-class histograms over the score space spanned by ED and CC scores coloured according to which class of solvent is most likely given the corresponding CC and ED scores. Water, sulfate, heterogen and metal classes are shown in blue, red, green and yellow, respectively. The dark blue and red regions correspond to contours containing 50% of observed water and sulfate in training data. Grey regions correspond to regions that are highly unlikely to correspond to a true solvent model.

The *PeakProbe* classifier entails three components: (i) a data-processing pipeline that maps 19 ED and two CC features to an ED/CC score pair, (ii) a solvent model prediction method that compares the relative likelihood with which this ED/CC score pair is associated with each of the four classes of solvent found in the training data and (iii) additional triage functionalities that address solvent–solvent interactions, variance in class frequency, model errors and peak clusters. Together, the ED and CC scores output by the data-processing pipeline form the basis of the two-dimensional score space shown in Fig. 2 ▸. Class distributions over this score space are modelled as two-dimensional histograms where each histogram bin encodes the likelihoods with which the ED and CC score values within the bin were observed for each of the four solvent classes considered. In Fig. 2 ▸, each histogram bin is coloured according to which solvent class is most likely given the ED and CC scores associated with that bin. Much like a Ramachandran plot, this histogram allows both visual and numerical evaluation of models given two independent inputs. To predict the most likely solvent model for a given peak, the *PeakProbe* classifier maps the ED and CC scores of the peak to a bin in this score space and then compares the likelihoods of each solvent class associated with this bin. The class with the greatest likelihood is selected as the predicted class of the peak. Class likelihoods are estimated from the joint ED/CC score distributions observed for each class in the training data. These distributions are weighted by various prior distributions, details of which are discussed below.

#### 2.2.1. Mapping feature data to ED and CC scores   

**Figure 3:**
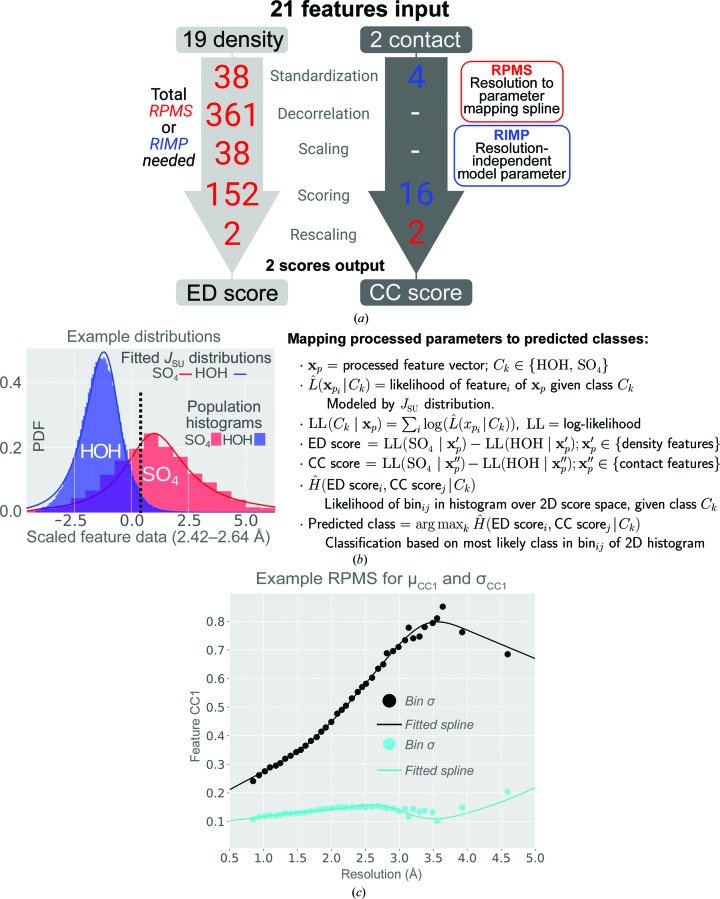
Data processing and feature scoring. (*a*) Procedures for mapping features to scores. For each group of features, the number of resolution-dependent parameters (red) or resolution-independent parameters (blue) required is shown. (*b*) Histograms of training data for a single feature in a single bin of resolution along with fitted probability density functions (PDFs) for water and sulfate data. Distributions like those shown are used to calculate ED and CC scores using the equations and definitions shown. (*c*) Example RPMS. Bin values for the observed mean (black dots) and standard deviation (cyan dots) for feature CC1 are plotted versus resolution. The spline fit to each series is shown as a solid line coloured similarly.

The data-processing pipeline of the *PeakProbe* classifier shown in Figs. 1 ▸ and 3 ▸(*a*) was designed to address the feature-versus-feature and feature-versus-resolution correlations observed in the training data described in Section 3. To construct a classifier that makes use of the discriminating ability of all 21 features described, we developed a data-processing pipeline that both standardizes and decorrelates all feature data in such a way as to yield feature values that are both independent of resolution and linearly independent of each other [Fig. 3 ▸(*a*)]. In this pipeline, ED feature values (CC1–CC14 and ED1–ED5) are standardized using resolution-dependent parameters. Standardized values are decorrelated by resolution-dependent principal component analysis (PCA) and scaled using additional resolution-dependent parameters. CC features (CF1 and CF2) exhibit minimal resolution-dependence or correlation and thus only undergo standardization using fixed, resolution-independent parameters.

Decorrelated ED feature data are converted to an ED score as follows: (i) the conditional likelihood that a peak corresponds to sulfate given a specific feature value is estimated from the probability density function (PDF) of the feature under consideration observed for all sulfate models in the training data, (ii) the parallel conditional likelihood estimate that a peak corresponds to water is calculated similarly, (iii) a joint conditional log-likelihood estimate that a peak corresponds to sulfate given all observed ED features is taken as the sum of log values of the conditional likelihood estimates obtained for each ED feature, (iv) the parallel joint log-likelihood estimate that a peak corresponds to water is calculated similarly and the resulting value is subtracted from that for sulfate to give a raw ED score, and (v) this raw ED score undergoes resolution-dependent scaling using resolution-dependent mean and standard deviation values to give the final ED score. CC feature data are converted to a CC score by an analogous procedure. For both the ED and CC scores, input feature values are scaled such that more positive score values represent more sulfate-like character.

To illustrate this scoring procedure, Fig. 3 ▸(*b*) shows the distribution of values for a single ED feature following decorrelation and scaling as observed for both water and sulfate populations in the resolution range indicated. These distributions are modelled as normalized histograms in blue and red, respectively. During classifier training, these distributions are modelled using a Johnson’s *S*_*U*_ (*J*_SU_) distribution, the resulting PDFs of which are shown as a solid lines (details are given below). The likelihood that a given feature value corresponds to sulfate or water is estimated from these PDFs. As an example, a value of 0.3 for this feature (dashed line) corresponds to likelihood estimates of 0.2 for sulfate and 0.05 for water and an estimated likelihood ratio of 4 for sulfate versus water. To calculate an ED score for a given peak, analogous likelihood values are estimated for each decorrelated and scaled ED feature. The corresponding likelihoods estimated from all decorrelated and scaled ED feature values are combined to give the raw ED score. Because the features used for likelihood estimation are transformed to a linearly independent basis by the preceding decorrelation procedure, individual likelihood estimates can be combined by assuming that their joint likelihood is given by the product of their individual likelihoods or, equivalently, the sum of their log values. Thus, the calculation of ED and CC score values as described finds many parallels in the construction of naïve Bayes classifiers and Fisher’s linear discriminant analysis.

#### 2.2.2. Class likelihood estimation   

Within the class likelihood estimation stage of the *PeakProbe* classifier, ED and CC scores are used to estimate the likelihood of each solvent class for a given ED/CC pair. This estimation takes advantage of the marked distinction between the joint ED/CC score distributions observed for each solvent class. As seen in Fig. 2 ▸, the ED/CC score distributions for each solvent class take on their maximum values over a distinct and continuous region of score space. During classification, models of the joint probability distributions for each solvent class are generated from the ED and CC distributions observed in the training data. ED and CC score distributions for each solvent class are modelled by discrete probability distributions and, because ED and CC scores are treated as linearly independent, their joint distribution is given by the outer product of the two distributions. The joint probability distribution of each class is weighted by a prior probability taken from one of three possible prior distributions (see below) and the likelihood that a given ED/CC score pair corresponds to a given solvent class is estimated. This estimate is given as the log of the ratio of the joint distribution value for a given class to the sum of the joint distribution values of the other three classes. For these estimates, a value of zero indicates that the likelihood of a particular score pair for a given solvent class is equal to that of all other classes combined. The more positive the estimate, the greater the likelihood that an observed score pair originates from a given class. When classifying a peak, *PeakProbe* selects the class with the greatest likelihood as the predicted class. The overall magnitude of the greatest class likelihood score is later incorporated into a quality indicator that reflects the confidence with which a given prediction is made.

#### 2.2.3. Peak triage and iterative peak classification   

In order for the classifier described above to provide accurate predictions of solvent models, several additional factors must be considered. Firstly, the *PeakProbe* classifier relies heavily on having a reliable estimate of the distance of a peak to the closest atom modelled in the structure (feature CF1), which cannot always be assumed to be the distance to the closest macromolecular atom. Thus, the classifier incorporates a mechanism for employing a peak–solvent distance rather than a peak–macromolecule distance when appropriate. Secondly, the predictions made by *PeakProbe* using the two-dimensional score space described vary according to the prior distribution of solvent classes used for likelihood estimation. However, not all structures exhibit the same relative frequency of solvent classes. Most notably, structures at coarser resolution typically contain fewer modelled waters than those at finer resolution. Thus, *PeakProbe* employs both fixed and adaptive prior weighting schemes during peak classification to allow for varied class frequency among structures and to provide a confidence indicator for each prediction made. Thirdly, because *PeakProbe* is designed to allow the prediction of solvent models at difference map peaks, the program employs a mechanism for distinguishing peaks that correspond to possible solvent positions from those arising from errors in the underlying structural model. Lastly, because difference map peaks often arise in clusters within large or extended ‘blobs’ of density, *PeakProbe* implements several graph-based clustering techniques to identify and characterize groups of peaks. These techniques are able to differentiate collections of peaks that are likely to be associated with a multi-atom solvent species from those likely to be constellations of water models or spurious peaks. These considerations are addressed in the triage stage of the *PeakProbe* classifier, details of which follow.

During feature extraction, CF1 is set to the distance from the peak to the closest macromolecular atom in the associated structure. However, many solvent species (water in particular) are anchored to the underlying macromolecular structure by hydrogen bonds to interstitial water or other solvent and not to the macromolecular components themselves. This phenomenon gives rises to the hydration shells observed in many structures and the periodic nature of the contact-distance distributions seen for water in protein structures (Biedermannová & Schneider, 2015 ▸). In such cases, where the distance from a peak to the nearest ordered solvent is shorter than the peak–macromolecular distance, using the longer distance results in an overestimated CF1 value. To overcome this problem, we incorporated a mechanism into *PeakProbe* for identifying peaks that are likely to be anchored by solvent contacts and update CF1 accordingly.

In order to extract reliable estimates of feature CF1, *PeakProbe* first identifies peaks with likely overestimated CF1 estimates and then searches the area surrounding the peak for plausible alternative contacts in closer proximity. Peaks with possibly overestimated CF1 values are identified by two criteria. Firstly, as seen in Fig. 2 ▸, water models typically have negative ED and CC scores, while heterogen models typically have low ED but positive CC scores. Because the CC score is highly correlated with feature CF1, peaks corresponding to water but with overestimated CF1 values will be misidentified as likely heterogen peaks. Thus, peaks with low ED and positive CC scores that are abnormally largely separated are flagged as having suspicious CF1 values. In addition, *Peak­Probe* calculates a probability that a given peak is water or not water based on the relative goodness of fit of the feature values of a peak to those observed in the training data. This score (C2; described in Appendix *A* [App appa]) allows further identification of suspicious CF1 values by identifying peaks with a high probability of being water but that are not predicted to be water by the classifier. To remedy possibly incorrect CF1 values, *PeakProbe* employs an iterative approach in which solvent molecules that are added or validated by *PeakProbe* are allowed to serve as anchors for peaks with suspicious CF1 values. If a peak–solvent distance is shorter than the previously set CF1 distance and the contacted solvent has been validated, the corresponding CF1 distance is updated. Peaks undergo the entire classification process following any CF1 updates, and newly assigned solvent molecules are evaluated as possible anchors for likely misclassified peaks. This process is iterated until no CF1 values are updated.

To account for the variability of solvent class frequency among structures, *PeakProbe* employs several prior weighting schemes for assigning likely models from ED and CC scores. As a whole, the PDB exhibits relative solvent class frequencies similar to those of the training data, which contain 96.2% water, 1.7% sulfate, 1.6% heterogen and 0.5% metal peaks. However, structures at coarser resolution contain a significantly smaller fraction of water, 76.1% of all structures contain no metal and only 3.2% of all structures contain all four classes. Thus, the use of a fixed prior distribution based on the relative class frequencies observed in the PDB may lead to distorted class likelihood estimates for structures with irregular solvent class distributions. As such, *PeakProbe* predicts likely solvent models using three different prior distributions. The first is a flat, uninformative, prior that weights each class equally (probability of 0.25). The second is the maximum-likelihood estimate for the distribution of occurrences of each class observed for structures in the training data (Dirichlet distribution; see Appendix *A* [App appa]). The third prior weights likelihood estimates by the class distribution observed for only the structure in which a peak resides. This distribution is constructed iteratively using high-scoring predictions from the *PeakProbe* classifier. Given a sufficient number of peaks, model predictions are made using the flat prior and a tally of high-likelihood predictions from each class is converted to a prior distribution. This updated prior is used to rescore peaks and the resulting distribution of predictions becomes the prior for additional rounds of scoring and prior updating. Ultimately, *PeakProbe* assigns a prediction to a given peak based on both the magnitude of and the degree of consensus among the likelihood estimates made by these prior weighting methods.

Attempting to use the classifier described above to evaluate peaks generated from local maxima in difference density maps encountered two critical problems that were not considered for peaks based on the coordinates of built and refined solvent models. Firstly, many observed difference map peaks corresponded to errors in the underlying macromolecular structure model, such as misplaced protein side chains or missing alternate conformations. For *PeakProbe* to predict solvent models for difference density peaks effectively, we reasoned that peaks arising from model errors or spurious map noise should be excluded from classification. Secondly, clusters of peaks in close association may correspond to a single underlying multi-atom solvent species and thus should evaluated as a group when predicting likely solvent models.

To avoid the inclusion of peaks arising from model errors as potential solvent models, *PeakProbe* implements a peak filter that takes advantage of several observations that were made following the trial classification of difference map peaks less than 2.0 Å from a macromolecular atom. The results from these trials showed frequent ED and CC score mismatches at peaks associated with macromolecular model errors. Specifically, ED scores tended to be high (more sulfate-like), while CC scores tended to be low (water-like). In addition, the number of short contact distances between the macromolecular structure and a putative water model fitted by real-space refinement tended to be anomalously high, even compared with metal ions, which show similar CC/ED score trends. Closer examination of test cases revealed that for many peaks associated with incorrect side-chain rotamers or backbone peptide geometry, a water model placed at the local difference density maximum moves towards the macromolecular model when refined against 2*F*_o_ − *F*_c_ density. Consequently, many clashes arise between this refined model and the underlying macromolecule, which is not included during feature extraction. *PeakProbe* employs both the mismatch in scores and the elevated count of clashing contacts to identify and filter out model error difference map peaks from those arising from unmodelled solvent.

To account for peak clustering, *PeakProbe* implements several graph-based techniques to identify groups of associated peaks, quantify the degree of association between grouped members and decide which peaks are diagnostic of any underlying solvent model and which should be considered satellites. These techniques make use of an adjacency matrix that both contains all difference map peaks for a given structure and accounts for adjacency arising from crystallo­graphic symmetry. Once constructed, this adjacency matrix is weighted by peak similarity and a combined divisive/agglomerative approach is used to identify and evaluate peak clusters. If a cluster of peaks is identified, peaks associated with the cluster are examined to determine whether any are predicted to be possible multi-atom solvent (sulfate or heterogen) based on the class likelihoods output by the classifier. If no peaks within a cluster are predicted to be multi-atom solvent, all cluster members are examined as possible water models. If one or more cluster member peaks is predicted to be multi-atom solvent, the most strongly predicted of such peaks is assigned as the cluster representative. The remaining cluster members are marked as either being (i) part of the same solvent model as the cluster representative, (ii) more strongly associated with the representative of a different cluster, (iii) a likely water model or (iv) an instance where a single-atom solvent model is associated with multiple peaks (a split peak). The fate of each peak within a cluster is determined by a combination of score values and the degree of graph similarity between the peak and the other cluster members. Ultimately, *PeakProbe* suggests multi-atom or water solvent models where appropriate for any input peaks found in close association (details are given in Appendix *A* [App appa]).

The *PeakProbe* classifier works most effectively when it is allowed to consider all possible locations for solvent models within a structure and when it is given as complete a macromolecular model as possible. In cases where a peak corresponds to a bona fide solvent model but the macromolecular structure associated with this model is missing, *PeakProbe* will not be able to calculate an accurate CF1 value. Furthermore, the extraction of ED features requires both 2*F*_o_ − *F*_c_ and *F*_o_ − *F*_c_ density maps, ideally with the *F*_o_ − *F*_c_ map calculated following the removal of all solvent models from the structure. Thus, *PeakProbe* can only be applied to structures with macromolecular models whose *F*_c_ coefficients provide a reasonable approximation of the total structure. In addition, both the peak-cluster analysis and adaptive prior weighting scheme described are most effective when given as much data as possible. If all solvent models are not removed from the structure prior to map calculation or the positions of likely solvent models are excluded from the input peak list, the cluster-analysis procedure may be missing data that are essential for correctly associating peaks with possible multi-atom solvent species. Likewise, the greater the number of solvent models predicted with high confidence by the classifier, the better the likelihood that the prior distribution generated from the tally of these predictions reflects the true distribution of solvent classes in the underlying structure. In any case, the *PeakProbe* classifier will output a prediction when given only 21 feature values and no other information, but such predictions will be less reliable than that those generated when *PeakProbe* analyses all likely solvent peaks within a structure in parallel.

### 2.3. Classifier training   

Classifier training entails determining the parameters that are needed to carry out each stage of the classification process shown in Fig. 1 ▸. During training, the parameters needed for each stage of classification are estimated from training data, and the training data are then subjected to this classification stage using these parameters in order to generate the data for the next stage. In total, classifier training estimates the over 4000 parameters that are needed to convert feature data to ED and CC scores, estimate class likelihoods, filter and flag peaks, and make model predictions. To train the classifier, the 21 features described were extracted from a total of 2 312 745 peaks taken from solvent models found in 17 274 structures ranging in resolution from 0.6 to 5.0 Å (training data; described in Section *A*1). Notably, owing to the extensive use of the vectorized array calculation capabilities of *NumPy* by *PeakProbe*, a complete training run using all training data requires less than 30 min using a single core of a single sixth-generation Intel Xeon CPU.

Within the *PeakProbe* classifier, the data-processing stage maps input features to ED and CC scores using a sequence of data transformations, each of which requires feature-specific and often resolution-specific parameters. Specifically, mapping the 19 resolution-dependent and highly intercorrelated ED features to an ED score involves five successive steps: (i) standardization of feature values, (ii) decorrelation of standardized values, (iii) scaling transformed values, (iv) scoring these rescaled values and (v) rescaling these scored values. In contrast, translation of the two resolution-independent and linearly independent CC values involves only the standardization, scoring and rescaling steps [Fig. 3 ▸(*a*)]. In this workflow, standardization refers to the conversion of raw feature values to *z*-scores using two estimated parameters for each feature (mean and standard deviation). Both scaling and rescaling are analogous to standardization, but instead of centring the resulting distribution of the training data at zero, the data are centred such that the midpoint between the means of sulfate and water populations is set to zero. As for standardization, scaling and rescaling require two estimated parameters per feature. Decorrelation involves the multiplication of an input vector of 19 standardized ED feature values by a 19 × 19 modal matrix whose coefficients are derived from the principal components of a data covariance matrix. Thus, decorrelation requires a total of 361 estimated parameters (19 per feature). Including a given feature in ED or CC score calculation requires the values of the observed sulfate and water PDFs of the feature to be obtained. During classifier training, these PDFs are modelled using Johnson’s *S*_*U*_ (*J*_SU_) distribution. This four-parameter distribution resembles a normal distribution, but allows arbitrary skew and kurtosis (Johnson, 1949 ▸). Modelling the PDFs for water and sulfate populations requires eight estimated parameters for each feature. In total, the conversion of 19 raw ED features to an ED score requires a total of 591 parameters.

Critically, each of the 591 parameters needed for ED score calculation varies strongly as a function of resolution. Thus, to classify a peak, *PeakProbe* requires a set of these parameters specific to the resolution of the peak. Rather than employ sets of parameters specific to given bins or ranges of resolution, we model each resolution-dependent parameter with a smoothly varying spline function [Fig. 3 ▸(*c*)]. When provided with a resolution value, these splines return a data-transformation parameter value for that resolution. We refer to such a function as a resolution-to-parameter mapping spline (RPMS). RPMS coefficients are determined by fitting six-parameter natural cubic spline functions to the training data using a moving-window approach. In this approach, feature values are binned by resolution and parameters are estimated from the data in each bin. The resulting set of parameter estimates is used along with the average resolution of each data bin to fit spline coefficients that parameterize the resolution-dependence observed for a given feature. The two example RPMSs in Fig. 3 ▸(*c*) show binned values for the mean and standard deviation of feature CC1 (circles) along with the spline functions used to model these data. Details of RPMS fitting, data decorrelation and distribution fitting can be found in Appendix *A* [App appa].

In contrast to the resolution-dependent parameters needed for ED feature mapping, the majority of parameters used for CC feature mapping are resolution-independent. Such parameters are simply global constants that are calculated during classifier training using all training data. We term these fixed parameters resolution-independent model parameters (RIMPs) to distinguish them from RPMSs. CC feature mapping requires two RIMPs for standardization and eight for modelling the water and sulfate PDFs for each feature. In total, CC feature mapping requires 20 RIMPs and two RPMSs, estimates for which are calculated during training using the same approaches as used for ED feature mapping parameter estimation.

Training the class likelihood-estimation stage of the *PeakProbe* classifier entails modelling the joint distributions of ED and CC scores observed for each solvent class in the training data. For each solvent class, ED and CC scores are binned and scaled to give discrete probability distributions. The values of these discrete ED and CC score distributions along with the data ranges used for score binning are the parameters generated during this training stage. Following this training stage, several additional parameters are derived from the training data, including the parameters that are necessary to convert C2 scores into probability estimates that a peak belongs to a class other than water. These probability estimates are used during the triage stage of classification and are incorporated into the quality indicator calculated for each prediction during classification.

The training process described provides the parameters that are needed to translate raw feature data into CC and ED scores. Many machine-learning techniques aim to produce similar linear projections of complex data, such as support-vector machines accompanied by ‘kernel tricks’ and isometric feature mapping-based manifold learning (Hofmann *et al.*, 2008 ▸; Singh *et al.*, 2007 ▸). Thus, it is reasonable to consider the system of data transformations parametrized by RPMSs/RIMPs used by *PeakProbe* as a means of obtaining a linear projection of feature data tailored to crystallographic data.

## 3. Results and observations   

### 3.1. Statistical properties and classifying power of individual features   

The utility of the 21 features described for distinguishing between water and sulfate was assessed by both a signal-to-noise metric and error analysis following classification using a logistic regression (LR) classifier constructed for each feature. The results of these analyses are given in Table 1 ▸, and details of the assessment metrics employed are detailed in Appendix *A* [App appa]. In Table 1 ▸, CC_r_ refers to the correlation coefficient of each feature versus resolution and S/N refers to a signal-to-noise metric that reflects the relative separation of feature-value distributions for water and sulfate models. Accuracy (Acc.) and *F*_1_ scores reflect the performance of a single-feature LR classifier in differentiating between sulfate and water. Accuracy is given by the fraction of class predictions that agree with the input labels. The *F*_1_ score is the harmonic mean of precision and recall, with precision defined as the fraction of sulfate predictions that are correct and recall defined as the fraction of input labelled sulfate that is correctly predicted. For highly unbalanced classes, the *F*_1_ score better accounts for false-positive predictions than does accuracy. To wit, for unbalanced populations in which waters (negative condition) outnumber sulfates (positive condition) by 50:1, similar to the training data described, predicting that all inputs are water results in an accuracy score of 98% but an *F*_1_ score of zero. Similarly, assigning classes at random from a 50:1 distribution results in a 50% expected accuracy but an expected *F*_1_ score of 3.8%. The stark differences between the accuracy and *F*_1_ scores for each LR classifier reflect the high false-positive rate (water models predicted to be sulfate) seen for all single-feature classifiers (Powers, 2011 ▸). Despite the high rates of false positives, all features were able to provide both *F*_1_ scores and accuracy rates in excess of random classification.

Further analyses revealed that the ability of each feature to distinguish between water and sulfate often varied strongly with resolution. Specifically, LR classifiers constructed and tested using training data from structures within a narrow resolution range often yielded markedly different *F*_1_ scores for different resolution ranges. For example, when trained on data with a resolution range of 1.0–1.3 Å, a logistic classifier using only CC13 yielded an *F*_1_ score of 0.4. When trained and tested on 3.3–3.7 Å resolution data, the resulting score was 0.8. *F*_1_ scores for the CC9 classifier exhibited a similar resolution-dependent performance, while CC5 exhibited the opposite trend, performing better at finer resolution. The resolution-dependence seen for the LR classifier performance of many features suggested that data for a given feature as a whole may not be linearly separable, but may be so piecewise when binned by resolution.

**Figure 4:**
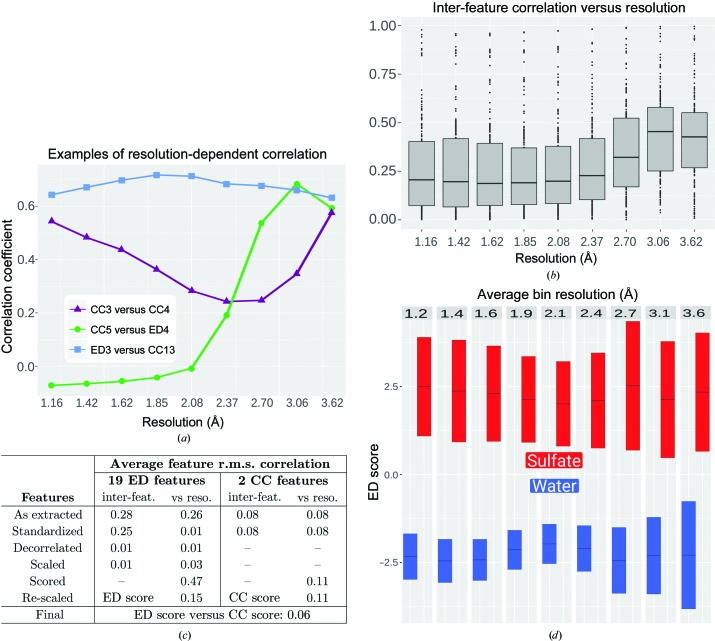
Results of data-processing methods on training data. In (*a*), (*b*) and (*c*), the values refer to Pearson’s product–moment correlation coefficient and r.m.s. refers to root-mean-square. (*a*) Inter-feature correlation for three example feature pairs. (*b*) Distributions of all 210 inter-feature correlation coefficients versus resolution. Coefficients are converted to r.m.s. values, plotted boxes correspond to values within the interquartile range, the median is shown as a horizontal bar and values outside this range are shown as dots. (*c*) Summary of inter-feature and versus-resolution correlation for ED and CC features at each stage of data processing. Inter-feature (inter-feat.) values refer to correlations between grouped features and versus-resolution (vs reso.) values to correlations between features and crystallographic resolution. Values are given for each stage in the data-processing workflow described in Fig. 2 ▸(*a*). (*d*) ED scores binned by resolution. Blue and red boxes represent the range of training-data ED scores for water and sulfate, respectively, that fall within one standard deviation of the mean (horizontal bar) of each resolution bin.

To investigate the implementation of a multi-feature linear classifier, the inter-feature correlations for each feature were analysed as a whole and for data binned by resolution. These analyses revealed that the majority of ED features show non-negligible inter-feature correlation and that the degree of correlation between feature pairs often varies with resolution. Fig. 4 ▸(*a*) shows the observed correlation coefficients for three feature pairs, two of which show marked changes in correlation across resolution ranges. Many inter-feature correlations were expected, such as the correlation of similar features extracted using both 2*F*_o_ − *F*_c_ and *F*_o_ − *F*_c_ density (for example CC3 and CC4). Fig. 4 ▸(*b*) illustrates the distributions of inter-feature correlation coefficients observed for training data. In this figure, feature data are binned by resolution and the distribution of correlation-coefficient magnitudes for all feature pairs is shown for each bin, where the median is shown as a horizontal bar, boxes correspond to values within the interquartile range and values outside this range are shown as dots. To construct a classifier that makes use of all extracted features simultaneously, we implemented a procedure for decorrelation by resolution-dependent PCA. When applied to training data, this decorrelation procedure disentangles the convoluted inter-feature relationships and resolution-dependence for all 19 ED features. Fig. 4 ▸(*c*) summarizes inter-feature and versus-resolution correlation for all features at each stage of data processing. Importantly, the average inter-feature correlation for ED features following decorrelation is essentially zero. Thus, even though complex and requiring thousands of parameters, this resolution-dependent data-processing pipeline successfully transforms raw feature values into linearly independent data suitable for use with linear classification methods, such as that used to calculate ED and CC scores.

### 3.2. Statistical properties and classifying power of ED and CC scores   

In addition to removing collinearity from feature data, the data-processing pipeline of the *PeakProbe* classifier produces ED and CC scores with minimal resolution-dependence. Fig. 4 ▸(*d*) shows ED scores by resolution bin for water and sulfate solvent classes as observed for the training data. For each class in each resolution bin, the coloured bar represents the range of ED scores ± one standard deviation from the mean (horizontal bar). For comparison, in Fig. 3 ▸(*c*) the mean of feature CC1 is plotted as a function of resolution along with a corresponding fitted RPMS. For this feature, the range of fitted values is 0.61, or 3.8 times the total standard deviation of all CC1 values. Calculation of this scaled range metric for all 19 ED features reveals a mean value of 3.2, indicating that on average the mean values for these features vary by more than three standard deviations when binned by resolution. In contrast, mean ED score values vary by only 0.52 standard deviations when binned by resolution. Thus, the resolution-dependent data processing described produces an ED score with a drastically reduced resolution-dependent variation when compared with the features from which this score is derived. CC scores show a similar behaviour, with minimal variation between resolution bins.

To investigate the ability of ED and CC scores to discriminate between different solvent classes, we repeated the analyses performed on each individual feature for each of these composite scores (Table 1 ▸). LR classifiers trained on ED and CC scores yielded *F*_1_ scores of 0.92 and 0.86, respectively, for distinguishing sulfate from water. Notably, these classifiers outperformed those trained on any single feature, indicating that each score successfully combines the classifying power of its constituent features.

Following up on this observation, we constructed the class likelihood estimator component of the *PeakProbe* classifier to assess the combined classifying ability of ED and CC scores. When training data for all sulfates and waters were input, this classifier achieved an accuracy of 99.98% and an *F*_1_ score of 98.05%. The *F*_1_ scores varied little with resolution, ranging from 95.9% at 2.70–3.10 Å resolution to 99.5% at 3.52–5.00 Å resolution. In order to investigate the impact of each individual feature on the combined classifier, we repeated these analyses using modified input data in which values for a given feature were swapped between the sulfate and water populations. In this approach, swapping data for any feature having a meaningful effect on ED or CC score values would result in a lower *F*_1_ score than observed for unmodified data. We define Δ*F*_1_ for a feature as the value by which the *F*_1_ score is lowered when values for the feature are swapped, and use this value as an overall indicator of feature impact (Table 1 ▸). The results of these analyses reveal that CF1, the distance from a peak to the closest model atom, and ED4, the volume of 2*F*_o_ − *F*_c_ density above 1.0σ, have the greatest effect on classifier performance, with both having Δ*F*_1_ magnitudes of greater than 0.8. Conversely, eight out of 21 features showed minimal feature impact, with Δ*F*_1_ magnitudes of 0.06 or less. However, all 21 features proved to have a measurable impact on classifier performance. We note that the probabilistic nature in which ED and CC scores are calculated results in self-weighting behaviour among input features. Specifically, by calculating ED and CC scores based on the relative likelihood of sulfate versus water for a given feature, for those features with minimal separation between these two populations (or at resolutions where such separation diminishes), the relative likelihoods of each population are nearly equal and their log ratio approaches zero. When feature likelihood log ratios are summed, features with near-zero values have no effect on the resulting score. Thus, each feature contributes to ED and CC scores only to the degree that it differentiates between populations in the training data.

**Figure 5:**
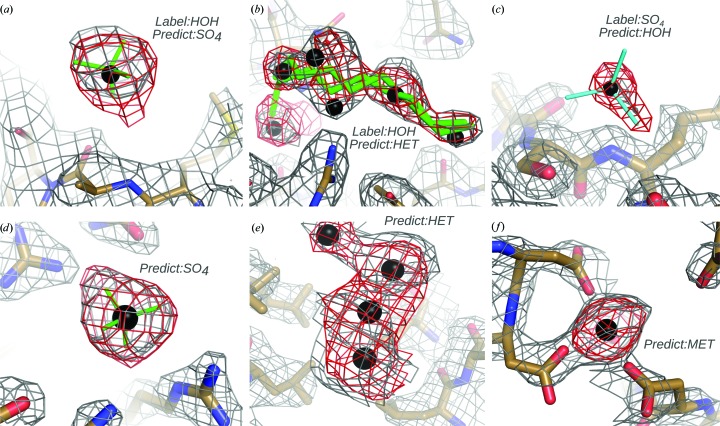
Peak-classification examples from six different structures. Peaks are shown as black spheres, macromolecular components are coloured by element (carbon in brown, oxygen in red, nitrogen in blue, hydrogen omitted) and posited models are shown in green. Electron density is shown as a mesh (*F*_o_ − *F*_c_ in red and 2*F*_o_ − *F*_c_ in grey contoured at 3.0σ and 1.0σ, respectively, unless noted otherwise). Top row: peaks from training data with nominally incorrect *PeakProbe* classifier predictions likely to be mislabelled in the PDB. Bottom row: peaks not associated with any existing solvent models but strongly predicted to belong to the class indicated by *PeakProbe*. Details are as follows. (*a*) PDB entry 4aqp (2.45 Å); the peak is water A2001 predicted to be a sulfate (modelled in green). Crystals were grown in the presence of the sulfate pseudo-analog 2-(*N*-morpholino)ethanesulfonic acid (MES). (*b*) PDB entry 2xrz (2.20 Å); the central peak is water B2012. Of the six peaks shown, four were strongly predicted to be heterogen. Crystals were grown in the presence of polyethylene glycol, a two-conformer model for which is shown as a point of reference in green. (*c*) PDB entry 2p3i (1.75 Å); the peak is the central S atom of sulfate A3000 (shown in cyan) and was strongly predicted to be water. (*d*) PDB entry 1mh3 (2.10 Å); the peak is adjacent to the terminal N atom of lysine A500 and was strongly predicted to be a sulfate (modelled in green). (*e*) PDB entry 2wjj (2.41 Å); four peaks are shown bracketed between the side chains of glutamate A95 and lysine A132, all strongly predicted to be heterogen. Crystallization conditions give no indications of likely models. (*f*) PDB entry 3zm4 (2.37 Å), *F*_o_ − *F*_c_ density contoured at 5.0σ, 2*F*_o_ − *F*_c_ density at 1.8σ; the peak is at a special position adjacent to aspartate A65 and is strongly predicted to be a metal. Crystals were grown in the presence of 0.2 *M* Ca^2+^ and the crystal lattice appears to be held together by electrostatic attraction between the acidic side chains shown and an unmodelled cation.

Further analyses revealed that the combined use of ED and CC scores allows the *PeakProbe* classifier to distinguish between solvent classes other than water and sulfate. For these analyses, class-versus-other *F*_1_ scores are tabulated for each class, with the smallest population assigned to the positive condition. The resulting *F*_1_ scores for each class are then averaged to give the reported score. Classification results data are given in Table 2 ▸. For training data, discrimination between only sulfate and water inputs resulted in an *F*_1_ score of 0.98, as noted above. Including all solvent classes and grouping all nonwater predictions into a single class resulted in an *F*_1_ score of 0.92. Classification with all four solvent classes yielded an *F*_1_ score of 0.82. Moreover, the *F*_1_ score of 0.82 belies a total accuracy of 99.1% and class-versus-other accuracy values above 98% for all solvent classes. Notably, omitting the CF1 updating and adaptive prior procedures during the triage stage of classification results in an *F*_1_ score of 0.48. For reference, when labels in the training set are randomly shuffled, the resulting predictions yield an *F*_1_ score of 0.02. Importantly, input labels in the training data were not subjected to any form of supervised validation and were taken as is. Thus, the training data certainly include label noise from incorrectly built models. The 98% classification accuracy observed for training data corresponds to a 2% total training error. Visual inspection of several hundred misclassified examples suggests that many such peaks are truly mislabelled and are not unique class instances mishandled by *PeakProbe*. Examples of likely mislabelled peaks are shown in Fig. 5 ▸. For the time being, we have elected to retain these data in the training set rather than risk introducing model bias through unsupervised data cleaning.

Because *PeakProbe* assigns scores based on unimodal distributions of feature values observed from large-scale sampling of the PDB, it seems unlikely that the probabilistic model derived from training data would be biased to include or exclude any particular subpopulation from any solvent class. Nonetheless, we assembled a validation data set of features from peaks that were explicitly not included in any training procedure to investigate possible model overfitting (detailed in Section *A*1). Overall, the classifier performance on the validation set mirrored that of the training data, with *F*_1_ scores of 0.96, 0.90 and 0.81 for sulfate/water only, water versus not water and all class classifications, respectively. The class distribution of misclassified validation data also resembles that of the training data.

### 3.3. Classifier performance on difference map peaks   

To test the effectiveness of the *PeakProbe* classifier on peaks derived from difference map maxima, a testing data set of features extracted from 69 817 difference density peaks associated with 2493 structures was assembled as described in Section *A*1. The performance of the trained classifier was evaluated using the same procedures as above, with overall results mirroring those obtained for training and validation data. Specifically, classification restricted to sulfate and water yielded an *F*_1_ score of 0.96, water versus not water gave a score of 0.90 and four-way classification scored 0.82, with class-versus-other accuracy scores above 99% for all classes. Table 3 ▸ details classifier performance using all four input and output classes for testing data. In the confusion matrix shown, each row corresponds to a single input class and each column to a single output class. Thus, diagonal elements correspond to correct predictions, while off-diagonal elements are errors. The *F*_1_ scores shown are calculated from the same classifier output after binning by resolution. For each class, the *F*_1_ score shown corresponds to a class-versus-other tabulation as used previously. These results show that *PeakProbe* is most successful at identifying water models, attaining class *F*_1_ scores for water classification above 0.92 across all resolution bins. *F*_1_ scores for other classes are lower than those seen for correctly predicting the water class, but are highly similar to the values seen for training data. As the binned *F*_1_ scores show, the ability of *PeakProbe* to differentiate between nonwater classes diminishes as the resolution worsens. However, the program is able to differentiate between these classes and water remarkably well even at unfavourable resolutions. These promising results suggest that *PeakProbe* performs equally well in classifying peaks derived from difference maps as it does for peaks taken directly from the coordinates of existing solvent models. Consequently, and as per our original objectives, the utility of *PeakProbe* extends beyond evaluating existing solvent models to providing model predictions for automated model building *ab initio*.

The ability of *PeakProbe* to predict highly likely solvent models for difference density peaks was further supported by an examination of the predictions made for unmodelled difference density peaks. When assembling and classifying the testing data, *PeakProbe* identified a total of 13 651 difference map peaks as strongly predicted to be solvent but not associated with any existing solvent model. Manual examination of several hundred such peaks revealed the vast majority of the *PeakProbe* predictions to be highly plausible. Examples of predictions for putatively unmodelled solvent are shown in Fig. 5 ▸. Table 3 ▸ shows the disposition of predictions for these peaks as ‘New solvent’.

As a preliminary test of the ability of *PeakProbe* to automatically build or rebuild solvent models, 277 structures used in the testing data set were selected at random and pipelined through *PeakProbe*. For each structure, the existing solvent models were removed, features were extracted from peaks generated from all difference density maxima above 3.0σ along with all modelled solvent atoms associated with these peaks, and all peaks were evaluated using the *PeakProbe* classifier. The solvent model output by *PeakProbe* was combined with the original solvent-stripped structure and the ensemble was refined with *phenix.refine* using three macrocycles of reciprocal-space coordinate and *B*-factor refinement. *PeakProbe* analysis was carried out both with difference map peaks and peaks from existing solvent together and with difference map peaks alone, ignoring input solvent models. In 62% of cases, the inclusion of the existing solvent model peaks for comparative evaluation resulted in lower post-refinement *R* factors than structures refined using solvent models built without comparison to existing models. In 82% of cases, refinement of the original structure with the solvent model generated by *PeakProbe* yielded lower *R* factors than those produced by the *ordered_solvent* procedure of *phenix.refine*, which provides automated water model-building functionality. In addition, refinement of the original structure with the solvent model output by *PeakProbe* improved the *R* factors compared with identical refinement of the original structure as deposited in 53% of cases. We stress that these last results are very preliminary and that the solvent models generated by *PeakProbe* have not been examined in detail. However, the observation that the inclusion of existing solvent models for comparison resulted in nominally higher quality *PeakProbe* models for a slight majority of structures indicates that further evaluation and development are needed for *PeakProbe* to predict and output quality solvent models in the absence of any existing solvent models for comparison.

## 4. Discussion and future development   

*PeakProbe* performs many of the functions associated with manually building a comprehensive solvent model for a macromolecular structure. Specifically, the program will (i) identify peak locations associated with possible solvent, (ii) extract features from these peaks, (iii) analyse these features by comparing them with features extracted from known solvent models and (iv) predict a likely solvent model for and evaluate any existing model already associated with the peak. Thus, *PeakProbe* serves as a prototype for fully automated solvent modelling. The approach taken by *PeakProbe* specifically addresses the gap in current software between tools for automated water modelling and those used for automated ligand identification and building.

At its core, *PeakProbe* predicts a likely solvent model for a given point in a macromolecular structure using both electron-density and local environment features. Predictions are made based on how features extracted from a given peak agree with the distributions for sulfate or water observed in a large-scale sample of the PDB. Although only water and sulfate are used as test models, the distinct trends in CC and ED features observed for other solvent classes allow the extension of the classes handled by *PeakProbe* to include the heterogen and metal classes. Constructing the *PeakProbe* classifier required the development of extensive data-manipulation routines for feature scaling and decorrelation to allow all extracted features to be incorporated into a linear classifier regardless of resolution. Obtaining a reasonable classifier performance using all four solvent classes required the inclusion of adaptive prior weighting to account for structure-to-structure variation in class frequencies and iterative updating of CF1 to allow solvent atoms to be anchored to other solvent atoms rather than directly to macromolecular atoms. The use of *PeakProbe* to evaluate difference map peaks required functionality for identifying both clustered peaks and peaks that were not associated with solvent models, such as those arising from errors in the underlying macromolecular models. *PeakProbe* incorporates routines to address all of these requirements and is able to classify difference map peaks and peaks based on existing models equally well.

The applicability of training data derived from existing models for classifying difference map peaks comes about as a direct result of the feature-extraction approach taken by *PeakProbe*. Specifically, all RSCC values are calculated after fitting fixed models into local density. For sulfate, starting from an idealized model and enforcing quality stereochemistry during refinement mitigates the effects of model errors such as distorted geometry or poorly refined *B* factors. Secondly, the refinement of both water and sulfate into density avoids the inclusion of RSCC scores from poorly fitted models in the PDB. Lastly, because difference map peaks do not directly correspond to optimal positions for likely solvent, models placed at these peaks require coordinate refinement, and thus the application of refinement during the acquisition of training data parallels the use of refinement needed for models placed at difference map peaks.

*PeakProbe* successfully maps feature data with resolution-dependent multi-collinearity to a resolution-independent vector space using a system of continuous regression models for resolution-dependent data-transformation parameters in the form of RPMSs. This approach can be viewed as the simultaneous application of spline regression and dynamic principal component analysis to produce a desirable data projection. These techniques find frequent use in a wide variety of data-analysis settings, but their combined use for facilitating the analysis of crystallographic data appears to be unique to *PeakProbe*. Moreover, the data-processing approach used by *PeakProbe* may find use in myriad situations in which data exhibit variant collinearity and dependence upon a continuous underlying variable similar to those observed for ED features.

While the performance of the *PeakProbe* classifier is highly accurate and the solvent models output by *PeakProbe* show promising results when used blindly in refinement, *PeakProbe* should not yet be considered a fully automated solvent-modelling tool. *PeakProbe* will carry over validated models for members of the heterogen and metal classes, but it cannot generate models for these classes *de novo*. Because *PeakProbe* considers sulfate and phosphate to be indistinguishable, models predicted to be sulfate should be converted to phosphate when appropriate. Furthermore, comprehensive testing of *PeakProbe* will require a thorough inspection of misclassified peaks leading to training error, classification error for existing solvent peaks outside the training data and validation of predictions for peaks that are not associated with any existing model. Until such testing is complete and a fuller extent of the capabilities and limitations of *PeakProbe* is known, predictions and models output by *PeakProbe* should be subjected to conscientious user validation. Given the sheer size of the training data, full remediation of putative mis­labelled inputs would require the examination of over 20 000 examples. Interestingly, similar large-scale crystallographic remediation efforts appear to be amenable to crowdsourcing approaches (Horowitz *et al.*, 2016 ▸; Jorda *et al.*, 2016 ▸), which is a possibility for *PeakProbe* that is currently under consideration.

## Figures and Tables

**Table 1:** Results of the analyses CC_r_ and S/N refer to correlation versus resolution and signal to noise, respectively. Acc. and *F*_1_ refer to accuracy and the *F*_1_ score, which assess classifier performance. Feature impact on classifier performance is measured by the Δ*F*_1_ metric and features are ranked (#) by this metric. Full descriptions of all terms are given in the accompanying text.

	Description					LR model		Feature impact	
Feature/score	Model	Map	Ref	CC_r_	S/N	Acc.	*F*_1_	#	Δ*F*_1_
ED features (real-space correlation coefficients)									
CC1	HOH	*F*_o_ − *F*_c_	Y	0.44	0.61	0.69	0.08	20	−0.04
CC2	HOH	2*F*_o_ − *F*_c_	Y	0.07	1.37	0.80	0.13	21	−0.03
CC3	SO4	*F*_o_ − *F*_c_	Y	0.51	4.70	0.92	0.30	16	−0.06
CC4	SO4	2*F*_o_ − *F*_c_	Y	0.10	9.09	0.97	0.52	12	−0.07
CC5	SO4 from CC3	[*F*_o_ − *F*_c_]^+^	N	0.36	0.31	0.66	0.06	10	−0.11
CC6	SO4 from CC4	[2*F*_o_ − *F*_c_]^+^	N	0.30	0.58	0.69	0.08	17	−0.05
CC7	SO4 from CC3	[*F*_o_ − *F*_c_]^+^	Y	0.38	0.36	0.69	0.07	8	−0.12
CC8	SO4 from CC4	[2*F*_o_ − *F*_c_]^+^	Y	0.31	0.64	0.71	0.09	14	−0.06
CC9	SO4 from CC7	*F*_o_ − *F*_c_	N	0.47	3.75	0.90	0.25	13	−0.07
CC10	SO4 from CC8	2*F*_o_ − *F*_c_	N	0.13	8.07	0.97	0.50	7	−0.15
CC11	HOH from CC1	[*F*_o_ − *F*_c_]^+^	N	−0.20	1.37	0.85	0.16	5	−0.16
CC12	HOH from CC2	[2*F*_o_ − *F*_c_]^+^	N	0.01	2.34	0.89	0.22	9	−0.11
CC13	Av. rotated CC3	*F*_o_ − *F*_c_	N	0.52	4.42	0.91	0.28	15	−0.05
CC14	Av. rotated CC4	2*F*_o_ − *F*_c_	N	0.15	9.27	0.97	0.54	11	−0.09
ED features (map values or derived)									
ED1	Standard deviation of CC13 values			−0.14	0.22	0.59	0.06	19	−0.04
ED2	Standard eviation of CC14 values			−0.08	0.19	0.48	0.05	18	−0.05
ED3	Peak *F*_o_ − *F*_c_ map volume			0.49	7.01	0.96	0.58	3	−0.74
ED4	Peak 2*F*_o_ − *F*_c_ map volume			−0.01	3.17	0.98	0.62	1	−0.83
ED5	Peak 2*F*_o_ − *F*_c_ map value (σ)			−0.15	0.88	0.84	0.14	4	−0.38
CC features									
CF1	Distance to closest model atom			0.14	5.24	0.97	0.56	2	−0.81
CF2	SO4-likeness of local environment			0.03	0.9	0.76	0.10	6	−0.15
Composite scores									
ED score	Mapping of 19 ED features			0.15	8.08	0.99	0.92		
CC score	Mapping of 2 CC features			−0.11	10.2	0.99	0.86		
C2 score	Pseudo-χ^2^ for ED/CC scores			0.14	3.50	0.96	0.46		
Total classifier	Maximum ED/CC likelihood			(2 classes) Acc. = 1.00, *F*_1_ = 0.98					

**Table 2:** Classification results For each trial shown, the models indicated by • were classified into the classes specified by ○, where W, S, H and M refer to water, sulfate, heterogen and metal, respectively. The S, H and M classes were taken as equivalent for ‘not water’. Accuracy and *F*_1_ scores are as in Table 2 ▸ (smallest population as the positive condition). The details of each data set can be found in Section *A*1.

	Input models				Scored classes					
Data set	W	S	H	M	W	S	H	M	Accuracy	*F*_1_ score
Training	2312745 solvent models from 17274 structures (0.6–5.0 Å)									
1	•	•			○	○			1.00	0.98
2	•	•	•	•	○	Not water			0.99	0.92
3	•	•	•	•	○	○	○	○	0.99	0.82
4	Randomized				○	○	○	○	0.92	0.02
Validation	89824 solvent models from 2573 structures (0.7–4.3 Å)									
1	•	•			○	○			1.00	0.96
2	•	•	•	•	○	Not water			0.99	0.90
3	•	•	•	•	○	○	○	○	0.99	0.81
Testing	69817 *F*_o_ − *F*_c_ > 3.0σ peaks from 2493 structures (0.7–4.5 Å)									
1	•	•			○	○			0.99	0.98
2	•	•	•	•	○	Not water			0.99	0.90
3	•	•	•	•	○	○	○	○	1.00	0.80

**Table:** (*a*) Confusion matrix.

	Predictions			
Labels	W	S	H	M
W	67965	3	32	32
S	85	389	22	1
H	233	45	443	6
M	101	31	15	414
New solvent	13334	84	155	78

**Table:** (*b*) *F*_l_ scores by class and resolution.

		Target class			
Resolution (Å)	No. of peaks	W	S	H	M
All	69817	0.99	0.89	0.75	0.76
0.70–1.31	4073	1.00	0.92	0.81	0.89
1.31–1.50	4919	0.99	0.80	0.74	0.65
1.50–1.72	14750	1.00	0.96	0.84	0.70
1.72–1.97	19018	1.00	0.94	0.77	0.79
1.97–2.25	15592	1.00	0.80	0.74	0.88
2.25–2.58	7793	1.00	0.89	0.71	0.82
2.58–2.95	2221	1.00	0.80	0.73	0.94
2.95–3.37	1226	0.96	0.86	0.65	0.61
3.37–4.50	185	0.92	0.56	0.67	0.51

## Appendix A Method and implementation details

### A1. Curation of data sets

For all data sets, a given data element consists of a PDB structure identifier, the coordinate at which feature extraction was carried out (a ‘peak’), the extracted features themselves and information about the local atomic environment surrounding the peak. Training data consist of all data used to derive the *PeakProbe* model parameters for classification and scoring. Two additional data sets were employed for testing the trained classifier. The first also uses PDB coordinates directly as peaks, while the second uses coordinates derived from local maxima within difference density maps calculated following the removal of solvent. These data sets are referred to as training, validation and testing data, respectively.

All training and validation data were assembled from features extracted from model atoms for each of four solvent classes: water, sulfate, heterogen and metal. Candidate models were collected from structures as follows: (i) selection of all PDB entries as of October 2016 accompanied by structure-factor data, not marked as obsolete and with a reported resolution between 0.6 and 5.0 Å, (ii) calculation of electron-density maps for each selected structure following the removal of water, sulfate and the 72 next most common single-atom and multi-atom solvent species in the PDB and (iii) discarding any structures with a working *R* factor of greater than 40% following the removal of solvent. Of the remaining structures, 18 256 contained sulfate or phosphate. From these structures, the central atoms of sulfate or phosphate with difference density above a nominal 3.0σ threshold were selected as candidate sulfate models, netting a total of 40 101 models from 12 333 structures. From these structures, all water atoms with difference density above 3.0σ were selected as candidate models (2 309 678 in total). Initial candidate heterogen and metal class models were selected from these same structures with the 3.0σ difference density requirement. Heterogen models were taken from the following molecules (PDB ligand codes given): glycerol (GOL), ethylene glycol (EDO), polyethylene glycol variants (PEG, PGE, PG4 and P6G), hexylene glycol (MPD), acetate ion (ACT) and chloride ion (CL). Candidate metal class models were taken from magnesium, calcium, zinc, cadmium, cobalt and manganese ions. To ensure sufficient populations within each class, heterogen and metal models from an additional 7422 structures not containing sulfate were included using the same *R*-factor and difference density selection criteria as noted above. This search yielded a total of 2 402 569 models from 18 815 structures including 6.7% and 50%, respectively, of all waters and sulfate/phosphates in the PDB. The authors note that all PDB entries were treated as independent structures when assembling data sets, including those sharing identical or highly similar macromolecular components, isomorphic crystal lattices and comparable limiting resolutions. Consequently, the data sets contain a small fraction of peaks that could be considered duplicate or redundant to the degree that identical solvent models in such overlapping structures would be associated with indistinguishable local electron density and atomic contacts.

From these data, all models from 1541 randomly selected structures were set aside as validation data, with the models from the remaining structures used as training data. For training purposes, heterogen class models were restricted to glycerol (GOL), polyethylene glycol (PEG) and acetate (ACT) and chloride ions only, and metal class models were restricted to magnesium, calcium, zinc and manganese. Metal and heterogen class models not included for training were placed with the validation data. All models are associated with a single atomic coordinate, either the atomic coordinate itself for single-atom species or that of a central atom for multi-atom solvent as follows (PDB atom names are given): SO4 (S), PO4 (P), GOL (C2), PEG (O2), ACT (C2). The final training data contained 2 312 745 models derived from 17 274 structures.

Validation data were assembled as follows: (i) from the 1541 structures previously set aside, all members of the aforementioned solvent classes were included as candidate models, (ii) multi-atom and metal species not included in the training data were included, (iii) candidate models were taken from coordinates for all atoms in multi-atom species provided that they were not the central atoms used in the training data and (iv) all models with difference density below 3.0σ were discarded. The testing data consisted of 89 824 models from 2573 structures, 1032 of which also contained separate models for training data.

Testing data were assembled from peaks identified in the difference maps calculated following the removal of common solvent. Difference density maps from the 2573 structures associated with testing data were subjected to a peak-search procedure that identified local maxima above 3.0σ positioned no closer than 0.3 Å but less than 8.0 Å from a macromolecular atom. This search netted 787 819 peaks, 374 563 of which could clearly be associated with existing solvent in the original structure. Class labels for these associated peaks were assigned based on the identity of the closest solvent atom, provided that it was less than 1.65 Å away and was among the species used previously for testing or training. Peaks associated with water models in the training data were discarded. The remaining peaks were filtered to remove those arising from model errors, map noise, satellite peaks of locally clustered peaks and those identified by *PeakProbe* as highly unlikely to correspond to solvent. Filtering procedures are described below. The resulting data set consisted of 69 817 difference map peaks associated with 2493 structures.

For all data, alternate conformations were treated as separate models for all classes. Electron-density maps were calculated using standard parameters for structure-factor weighting and bulk-solvent scaling in *PHENIX* (Adams *et al.*, 2010 ▸).

### A2. Feature extraction

All electron-density features were collected by centring peaks in a reference coordinate system consisting of a 10 Å triclinic cubic cell, resampling the accompanying electron density in this cell using tricubic interpolation with 0.5 Å lattice spacing and fitting models for both water and sulfate by real-space refinement. For sulfate models, the central sulfur was centred within the reference cell, with each bonded O atom placed 1.51 Å away with idealized tetrahedral geometry (Miehlich *et al.*, 1989 ▸). Standard geometric restraints from the *cctbx* libraries were used along with an automatically weighted harmonic restraint on the S atom. Water models were refined as point atoms (no explicit H atoms) with a similar harmonic restraint. The use of this reference setting greatly facilitates program speed because a single set of input models and geometry restraints can be used for refinement against any target density.

For features CC1–CC14, real-space correlation coefficient (RSCC) values were calculated for a given model against a given map as described in Table 1 ▸. The volume of density associated with each peak (ED3 and ED4) is given by the number of grid points in the reference setting with density above 1.0σ within 2.0 Å of the initial position of each peak. Density values were corrected for the effects of variance underestimation owing to solvent flattening. To capture the radial dependency of the local electron density, we employed a ‘pseudo-inverse’ electron-density map (denoted with a ‘+’ in Table 1 ▸) where each scaled electron-density value is mapped to an approximate multiplicative inverse value. This pseudo-inverse is constructed so as to asymptotically convert large or negative output values to a fixed maximum as input values decrease. Use of this map effectively upweights density farther from the peak centre, thus upweighting the region of density occupied by sulfate O atoms but relatively unoccupied by water. To quantify the tetrahedral character of sulfate, two sets of RSCC values were calculated following two coordinate transformations. Firstly, a sulfate model was refined against the pseudo-inverse map and the resulting coordinates were used for RSCC calculation against the original, non-inverted map (CC9 and CC10) with the expectation that fitting a sulfate model to pseudo-inverse density (restraining the central S atom) would place the O atoms maximally out of place with respect to the non-inverted density. Secondly, a sulfate model was first fitted to a given map and the model was then rotated by 60° around a bond axis so as to explicitly move three of the four bonded O atoms out of phase with any tetrahedral morphology within the observed density. In both cases, the coordinate transformation would result in a commensurately poor RSCC for true sulfate density, but a relatively unaffected RSCC for true water density. For refinement transformations, RSCC values from non-inverted maps were used directly as features. For the explicit transformations, the rotation operation was repeated for all four sulfate bond axes with average values (CC14 and CC14) and standard deviations (ED1 and ED2) used as features.

Close-contact features were obtained from the analysis of modelled atoms within 6.0 Å, including those arising from crystal symmetry. Atomic identities and interatomic distances for all peak-atom pairs were found using *cctbx.fast_pair_generator*. Feature CF1 is the distance from a peak to the closest modelled atom. This atom can be considered as the ‘anchor’ that associates a putative solvent molecule at the peak position with the structure model as a whole. Feature CF2 quantifies the relative likelihood of sulfate versus water given the other atoms in the vicinity.

To obtain CF2, we tabulated the frequency with which sulfate and water occur within 5.0 Å of every chemically unique atom observed for either population. The list of chemically unique atoms is the set of concatenated atom and residue name PDB records, and only instances within the training data for water or sulfate central atoms with difference map density values of greater than 5.0σ were tabulated. Observed frequencies were normalized to give the frequency with which water or sulfate is found in proximity to a given atom. The ratio of these frequencies for sulfate versus water for observations with at least 100 total occurrences for both populations were calculated. A table of log values of these ratios was then used to score the overall ‘sulfate-likeness’ of the surrounding environment. This score was determined by finding all atoms with table entries within 5.0 Å of a given peak (alternates merged) and summing their respective log-ratio values to give CF2. We note that no distance weighting was applied during tabulation: the observed frequency log ratio for a given atom was included in the score if the atom was within 5.0 Å of the peak regardless of distance. Applying either 1/distance or 1/distance^2^ weighting did not substantially alter the ability of CF2 to discriminate between solvent classes.

The C2 score represents the probability that a given peak is water or not water based on the relative goodness of fit of the feature values of a peak to those observed in the training data. This score was calculated by first taking the squared differences between observed feature values and the mean values of the *J*_SU_ distributions fitted for each feature and class. Each squared difference was divided by the variance of each distribution and the resulting values were summed for both sulfate and water distributions. Because *J*_SU_ distributions are similar but not identical to normal distributions, these values are similar but not identical to a χ^2^ statistic, although their observed distributions from training data are well fitted by χ^2^ distributions and thus are termed pseudo-χ^2^ values. The pseudo-χ^2^ value for features versus observed water distributions was subtracted from that of the sulfate distributions and the resulting value was converted to a C2 score by a two-parameter logistic function. Parameters for this function were obtained from all training data using a two-class model (water versus not water).

### A3. Peak-cluster analysis

During feature collection, *PeakProbe* assembles a list of all peaks within 6.0 Å of a given peak along with the corresponding peak–peak distance and symmetry operation. To fully encapsulate the relationships between all peaks, including those arising from crystal symmetry, a rank-4 (*n*, *n*, *s*, *s*) multi-layer adjacency tensor (MLAT; De Domenico *et al.*, 2013 ▸) is constructed where *n* is the number of peaks within a structure and *s* is the number of symmetry operations encountered during the peak–peak distance search (not necessarily the number of symmetry operations associated with the space group of the underlying structure). This MLAT can be thought of as a system of *s* ^2^ adjacency matrices, each *n* × *n*, where the two *s* indices represent enumerated asymmetric units in which the peak–peak contact (edge) originates and terminates. For peak clustering, the MLAT is undirected and layers with identical *s* indices are identical recapitulations of contacts within a single asymmetric unit. Contacts generated by symmetry are represented in layers where the *s* indices are not equal. When considering only a single asymmetric unit, only *s* = 1, *i* where *i* = {1, 2, …, *s*} layers need to be considered. Following the construction of a complete MLAT, a shortest-path search was carried out for all *i* across an above-adjacency matrix constructed from the 1, *i* layers of the MLAT. For every peak–peak pair, this search provides a list of graph-traversal distances between the two peaks making use of, at most, a single symmetry-related asymmetric unit. These distances were folded back into a single asymmetric unit by constructing a distance-weighted peak–peak adjacency matrix in which each matrix element is the shortest peak–peak distance observed for each pair.

Following the construction of this modified adjacency matrix, a divisive approach was used to identify closely associated peaks. From the adjacency matrix, a similarity matrix was constructed using a density-weighted metric incorporating the similarity in the CC, ED and C2 scores and the local density between connected nodes. This similarity matrix was converted to a minimum-cut tree by successive spectral partitioning using Fiedler vectors of each subgraph. All peak–peak connections were ranked by density-weighted similarity. Starting from the most strongly connected peak pair, peak clusters were built up by single-linkage agglomeration of candidate cluster members drawn from the smallest cluster in which both peaks reside in the minimum-cut tree. For each resulting cluster, the cluster score is the average pairwise similarity (edge weight) for all cluster members. Peaks occurring in multiple clusters were removed from all but the cluster with the highest cluster score and peaks within a cluster were ranked by degree centrality. Using features collected from water and all atoms of multi-atom solvent from 1000 test structures, the cluster score described is able to differentiate water clusters from multi-atom solvent with 92% precision.

### A4. Feature and classifier evaluation

For the features and scores described in Table 1 ▸, the correlation of each feature or score with crystallographic resolution (CC_r_) is the Pearson product–moment correlation coefficient for the two variables. Logistic regression (LR) classifiers were constructed by fitting a regularized, unweighted two-parameter logistic target function to standardized feature values from a balanced sampling of water and sulfate from the training data. Sampling and parameter estimation were repeated until the average value for each parameter varied by less than 0.1%. The performance of each feature or classifier was assessed by tabulation of accuracy and *F*_1_ scores with sulfate as the positive condition. Accuracy is the fraction of all predictions that match input labels. The *F*_1_ score is the harmonic mean of precision and recall, with precision defined as the fraction of sulfate predictions that were correct and recall defined as the fraction of input labelled sulfate that was correctly predicted. For evaluation of multi-class classification, class-versus-other *F*_1_ scores were tabulated for each class, with the smallest population assigned to the positive condition. The resulting *F*_1_ scores for each class were then averaged to give the reported score.

The overall effect of each feature on the *PeakProbe* classifier (feature impact) was assessed by swapping feature data for peaks labelled as sulfate for data from peaks labelled as water and *vice versa*, followed by recalculation of the *F*_1_ scores. To accommodate the unbalanced sizes of each class, this swapping was performed by assigning values for one class from a random sampling (with replacement) of data from the other class. Starting with all sulfate and water training data correctly predicted by the *PeakProbe* classifier (*F*_1_ = 98.0%), data for a given feature were swapped as described and the altered data were mapped to ED and CC scores. Predictions from these scores were used to calculate new *F*_1_ score values, and the resulting change in the *F*_1_ score (Δ*F*_1_) reflects the impact of swapping class data for a given feature, and thus indicates the net effect that a given feature has on classifier performance.

### A5. Model training

#### A5.1. Modelling of resolution-dependent parameters by RPMSs

All resolution-dependent parameters were modelled using six-parameter natural cubic spline functions. Given a resolution value, these fitted spline functions evaluate a best-fit estimate for a given parameter and thus are termed resolution-to-parameter mapping splines (RPMS). The use of such spline functions ensures that parameters vary smoothly with resolution and reduces the number of training parameters that are needed to encapsulate feature distributions over the entire range of observed data. To obtain RPMS coefficients, data were binned by resolution and the estimated parameter and the mean resolution of each bin were fitted using the BFGS minimizer of *SciPy* using an *L*_1_-norm regularized squared-error target function. For certain parameters, fitted splines tended to take on extreme values at resolutions outside the observed data. To counteract this behaviour, data points at the highest and lowest (numerically) resolution were duplicated at 5.0 and 0.3 Å, respectively, prior to fitting. The natural spline bases used impose linear behaviour outside the terminal spline nodes (fixed at 1.0 and 4.0 Å) and this data augmentation, albeit artificial, allows reasonable parameter extrapolation to extreme resolutions (D. Madigan; http://www.stat.columbia.edu/~madigan/DM08/regularization.ppt.pdf).

For all RPMSs, splines were fitted following sorting of all data by resolution and grouping data into bins of sufficient resolution-spanning width to ensure that each bin contains a sufficient sample size. For resolution-dependent mean parameters, averages for the resolution and parameter value for each bin were fitted directly. Standard deviation values were converted to log values prior to fitting, ensuring that the anti-log of the fitted value was positive. For modal matrix elements and distribution coefficients, a moving-window approach was used in which a number of contiguous bins were grouped and this grouping was shifted through the data bin by bin as average parameter and resolution values were accumulated.

#### A5.2. Decorrelation of ED features

Resolution-dependent decorrelation was accomplished by transforming data via principal component analysis (PCA). The required transformation matrix was constructed from a unitary matrix obtained from singular-value decomposition of the Pearson product–moment correlation matrix. The left-singular vectors (eigenvectors) of the resulting decomposition were sorted according their singular values and were arranged as columns to form a modal matrix. To obtain resolution-dependent modal matrix elements (i) the correlation matrix for all data was decomposed and the resulting unitary matrix was arranged to give a reference modal matrix, (ii) similar unitary matrices were calculated for data over a narrow resolution range using the moving-window approach described, (iii) the left-singular vectors of each of these matrices were permuted so as to maximize agreement with a reference matrix and (iv) values for each matrix element and the average resolution of the data window were modelled by RPMS. The vector permutations used consist of column rearrangements and additive inversion. The orientation of the vector corresponding to the smallest singular value was oriented so as to ensure that the determinant of the resulting modal matrix is 1.0. During the vector-permutation process, vectors from the unitary matrix derived from the window of data with the lowest numerical resolution were compared with the reference modal matrix. Vectors from subsequent data windows were compared with the modal matrix from the previous window of data. Training data were divided into 71 resolution bins and were analysed using a five-bin moving window.

#### A5.3. Modelling of observed distributions of feature values

To model the conditional likelihood distribution of a given feature for a given class (sulfate or water), a normalized histogram of observed data for each sulfate and water population was fitted to a *J*_SU_ probability distribution. For resolution-independent CC features, training data for both all waters and all sulfates were fitted directly. For resolution-independent ED features, data were binned and windowed as described for decorrelation, and histograms of data from each window were used for fitting. The sparsity of data at high resolution resulted in a few irregular histograms that were refractory to distribution fitting. In addition, *J*_SU_ parameters exhibit highly correlated behaviour in certain regions of parameter space. To generate fits under these circumstances, initial fit values were taken from the immediately preceding resolution bin and the fitted values were restrained to these initial values by including harmonic terms in the fit target function. Further fit conditioning was accomplished by including an *L*_2_-norm for two of four parameters and a penalty on the variance of the resulting distribution. Example fits are shown in Fig. 4 ▸(*c*). The slight systematic errors in the fit are attributable to the specific parameter constraints that were applied during fitting. Lastly, for a given solvent class, the continuum of *J*_SU_ distributions for resolution-dependent feature is itself modelled by fitting an RMPS for each distribution parameter.

#### A5.4. Modelling of joint ED/CC and class frequency distributions

Observed joint probability distributions for each solvent class given ED and CC scores were constructed using normalized histograms of each score. The score data were divided into 50 equal-width bins and the frequency of scores occurring within each bin along with the bin centroid were stored as model parameters. Each structure in the training data contains a distribution of the four included solvent classes. The distribution of these distributions across all structures in the training data constitutes a Dirichlet distribution conjugate to the categorical posterior distribution of classes. Thus, the most likely class distribution based on this Dirichlet distribution constitutes an appropriate prior for class weighting. The maximum-likelihood estimate for the parameters of this distribution was calculated using the set of observed class probability mass functions (with additive smoothing) for all structures in the training data (J. Huang, http://jonathan-huang.org/research/dirichlet/dirichlet.pdf). The expected values of these parameters were used to construct the final prior distribution used for class weighting.

### A6. Software notes

All Python modules used and required for classification are incorporated into the *cctbx.python* environment. Model training makes use of *SciPy* modules, several of which are incompatible with *cctbx.python*; thus, model training must be run outside this environment. All algorithms described are implemented using *NumPy*. Statistical analyses were carried out using *NumPy*, *R* and *gnuplot* (Williams & Kelley, 2013 ▸; R Development Core Team, 2008 ▸). Structure figures were prepared using *PyMOL* (v.1.504; Schrödinger).
